# Hidden genomic diversity drives niche partitioning in a cosmopolitan eukaryotic picophytoplankton

**DOI:** 10.1093/ismejo/wrae163

**Published:** 2024-08-14

**Authors:** Yangbing Xu, Shara K K Leung, Taylor M W Li, Charmaine C M Yung

**Affiliations:** Department of Ocean Science, The Hong Kong University of Science and Technology, Hong Kong SAR; Department of Ocean Science, The Hong Kong University of Science and Technology, Hong Kong SAR; Department of Ocean Science, The Hong Kong University of Science and Technology, Hong Kong SAR; Department of Ocean Science, The Hong Kong University of Science and Technology, Hong Kong SAR

**Keywords:** eukaryotic phytoplankton, biogeography, genomic diversity, adaptation, climate change

## Abstract

Marine eukaryotic phytoplankton are fundamental to the marine food web, yet the lack of reference genomes or just a single genome representing a taxon has led to an underestimation of their taxonomic, adaptive, and functional diversity. Here, we integrated strain isolation with metagenomic binning to recover genomes from the cosmopolitan picophytoplankton genus *Bathycoccus*, traditionally considered monospecific. Our recovery and analysis of 37 *Bathycoccus* genomes delineated their global genomic diversity and established four evolutionary clades (BI, BII, BIII, BIV). Our metagenomic abundance survey revealed well-differentiated ecological niches and distinct biogeographic distributions for each clade, predominantly shaped by temperature, salinity, and nutrient availability. Comparative genomics analyses further revealed clade-specific genomic traits that underpin niche adaptation and contribute to the global prevalence of *Bathycoccus*. Our findings underscore temperature as a major driver of genome diversification in this genus, with clade divergences coinciding with major paleoclimatic events that influenced their contemporary thermal niches. Moreover, the unique enrichment of C2H2 zinc finger and ankyrin repeat gene families in polar-adapted clades suggests previously unrecognized cold-adaptation mechanisms in marine eukaryotic phytoplankton. Our study offers a comprehensive genomic landscape of this crucial eukaryotic picophytoplankton, providing insights into their microdiversity and adaptive evolution in response to changing environments.

## Introduction

Eukaryotic phytoplankton, highly diverse photosynthetic microorganisms, are pivotal to primary productivity and global biogeochemical cycles within marine ecosystems [1]. The coexistence of numerous phytoplankton species within marine habitats and the ecological mechanisms shaping their distribution represent fundamental and long-standing enigmas in microbial oceanography [2, 3]. Understanding the complex patterns and determinants of biodiversity and biogeography is crucial for elucidating the ecological dynamics of phytoplankton and their resilience to environmental changes, thus highlighting the need for comprehensive genomic information of these organisms. Compared with prokaryotic genomes, eukaryotic genomes typically larger and more complex, replete with introns, pseudogenes, and repetitive elements [4]. These features, compounded by challenges in isolation and cultivation, have impeded the acquisition of eukaryotic genomes, thus delaying the exploration of eukaryotic phytoplankton genomes from natural communities relative to prokaryotes.

Although 16S/18S rRNA gene amplicon sequencing has made significant strides in uncovering previously unknown groups within the uncultured microbial majority [5, 6], the genomic clades with high marker gene sequence similarity (>97%, or even >99%) within microbial populations, being regarded as “microdiversity” [7, 8], has only been largely recognized due to the advances in genome-resolved analyses. The findings from these analyses have challenged the traditional notion of a single “species”, revealing instead that what was once considered a single species can actually be divided into multiple “genospecies” [7, 8]. The microdiversity is prevalent in prokaryotic phytoplankton, where diverse genospecies correspond to distinct ecotypes, each with unique biogeographic distributions and functional traits [9–11]. Although this microdiversity has been evident in several well-studied group, such as *Gephyrocapsa huxleyi* [12], the paucity of reference genomes for most eukaryotic phytoplankton taxa has left their genomic diversity poorly defined. This knowledge gap poses a risk of underestimating their adaptive and functional diversity, which is crucial for understanding fine-scale niche partitioning and predicting shifts in phytoplankton communities under changing ocean.

Recent advancements in metagenomic technologies have revolutionized the study of uncultured eukaryotic phytoplankton by enhancing genome assembly and binning techniques. These improvements have facilitated the large-scale reconstruction of genomes from various eukaryotic lineages, expanding our knowledge of how environmental factors influence their genomic diversity [13–15]. Eukaryotic genomes from groups with substantial biomass and streamlined genomes have been preferentially assembled, resulting in higher-quality reconstructions [13–15]. In particular, Mamiellophyceae, a class of green algae, is one of the most frequently encountered taxonomic groups in genome recovery efforts from the euphotic zone. Thus, the metagenome-assembled genomes (MAGs) provide deep insight into the global genomic landscape of these dominant eukaryotic phytoplankton.

The Mamiellophyceae, comprising the three major genera, *Ostreococcus*, *Micromonas*, and *Bathycoccus*, represents ecologically important groups of marine eukaryotic picophytoplankton (with cell diameter of 0.6 to 3 μm). These unicellular organisms are globally distributed and are the predominant component of the picoeukaryotic biomass in coastal waters [16–18]. They are culturable and possess streamlined genomes from 13 to 21 Mb, making them valuable models for investigating ecological and evolutionary processes in eukaryotic phytoplankton [16]. *Bathycoccus*, in particular, showcases remarkable adaptation across diverse environmental gradients, from tropical to polar regions [19, 20]. Traditionally, the classification of *Bathycoccus* was constrained to a single species, *Bathycoccus prasinos*, as defined by the 18S rRNA gene biomarker. However, recent genomic discoveries have now unveiled *Bathycoccus calidus* as a distinct species, revealing a previously underestimated species richness and ecotypic diversity within the genus [20, 21]. Despite these advancements, the majority of genomic studies on *Bathycoccus* have focused on oceanic waters, with other environments such as brackish and estuarine waters remaining under-investigated. This oversight suggests that the complete genomic diversity of *Bathycoccus* on a global scale has yet to be fully documented. A more comprehensive analysis of the genome diversification of *Bathycoccus* and its interactions with environments could elucidate the mechanisms underlying its ecological success and provide deeper insights into the microdiversity and niche adaptation within eukaryotic phytoplankton.

This study combines strain isolation and metagenomic binning techniques to acquire a diverse array of *Bathycoccus* genomes from oceans worldwide. Through in-depth analysis and comparison of these genomes, we aim to: (i) elucidate the global genomic diversity and phylogeny of *Bathycoccus*; (ii) identify the environmental factors that drive their diversification and distribution; and (iii) uncover the genomic adaptations that enable their survival across various habitats, ultimately contributing to their remarkable global distribution*.* These findings will enhance our understanding of the fundamental questions of biodiversity and biogeography among eukaryotic phytoplankton, as well as their response to ongoing changing climate.

## Materials and methods

### Strain isolation, identification, and cultivation

*Bathycoccus* strains were isolated from surface seawater samples collected across Hong Kong from 2020 to 2022 (Fig. S7). Samples were filtered using 0.6, 0.8, or 1 μm polycarbonate filters (Sterlitech, USA), mixed with L1 medium, and incubated at 20°C under a 12:12 h light–dark cycle at 30 μmol m^−2^ s^−1^ light intensity. The grown algae were transferred to fresh L1 medium every 2 weeks. Algal DNA was extracted for PCR targeting the V4 of 18S rRNA gene and ITS1-5.8S-ITS2 regions to identify strains [22], with positive *Bathycoccus* samples retained for further research (Table S1). Strains were purified using serial dilution and antibiotic treatments (Table S1).

### Nucleic acid extraction, sequencing, genome assembly, and annotation

We selected the *Bathycoccus* strain UST710 for whole-genome sequencing. Details of nucleic acid extraction and sequencing, genome assembly, annotation of repetitive elements, endogenous viral elements (EVEs) identification, gene prediction, and functional annotation are provided in Methods S2.

### Reconstruction of *Bathycoccus* genomes from public datasets

To explore the global genomic diversity of *Bathycoccus*, we downloaded and analysed marine metagenomic samples from public datasets, focusing on understudied regions such as South China Sea (SCS) (Table S9). Raw metagenomic reads were trimmed using Trimmomatic v.0.39 [23] and assembled using MEGAHIT v.1.2.9 [24] with default parameters, either individually or collectively (Table S5). Contigs over 1500 bp from each assembly were binned using MetaBAT v.2.0 [25] and their quality was assessed using BUSCO v.5.2.2 [26] and EukCC v.2.1.0 [27], retaining bins with >50% completeness and <2% contamination. Besides, we compiled *Bathycoccus* genome resources (MAGs and SAGs), from published datasets and evaluated their completeness and contamination to exclude unqualified genomes. In total, we acquired 37 qualified *Bathycoccus* genomes, including a new strain UST710 (Table S6). We used AUGUSTUS v3.4.0 [28] with the training species model of “*Bathycoccus prasinos*” to predict functional genes for these genomes. The rRNA gene and ITS regions in genomes were annotated using Barrnap v.0.9 (https://github.com/tseemann/barrnap) and ITSx v.1.1.3 [29], respectively.

### Phylogenetic analyses

Phylogenetic analyses were performed using the ITS1-5.8S-ITS2 sequences from isolated Hong Kong strains, metagenomic assemblies MAGs, and NCBI GenBank (Table S8), with a maximum-likelihood (ML) tree was constructed using IQ-TREE v.2.2.6 [30] under the K2P + I + G4 model, with 1000 ultrafast bootstrap iterations. The secondary structures of the ITS2 sequences were predicted using RNAfold (http://rna.tbi.univie.ac.at/cgi-bin/RNAWebSuite/RNAfold.cgi). OrthoFinder v.2.5.5 [31] was used to cluster proteins of the 37 qualified *Bathycoccus* genomes, along with *Micromonas* and *Ostreococcus* reference genomes, into orthologous gene groups. An ML phylogenomic tree was constructed using concatenated alignments of these single-copy orthologs with IQ-TREE v.2.2.6 [30] under the Q.pfam+F + I + R5 model, with 1000 ultrafast bootstrap iterations. Both trees were visualized using tvBOT [32]. Additionally, pairwise average nucleotide identity (ANI) and average amino acid identity (AAI) among the 37 qualified *Bathycoccus* genomes was calculated using FastANI v.1.33 [33] and EzAAI v1.2.3 [34], respectively.

### Biogeography of different *Bathycoccus* clades

Metagenomic reads were aligned to representative genomes of four *Bathycoccus* clades (BI: *B. prasinos* RCC1105; BII: TARA_ION_45_MAG_00030, MAG; BIII: *Bathycoccus* sp. UST710; BIV: ERR2206775_bin.1, MAG) using the bbsplit.sh script (https://jgi.doe.gov/data-and-tools/software-tools/bbtools/), with parameters of “minratio=0.99 ambiguous=all ambiguous2=split”. Ambiguous reads mapping to multiple references were excluded. Metagenomic dataset details are in Table S9. Relative abundances were normalized to RPKM (reads per kilobase per million mapped reads). Canonical correlation analysis (CCA) was performed using the OmicShare tools (https://www.omicshare.com/tools) to illustrate the associations between environmental parameters and the abundance of different *Bathycoccus* clades.

### Growth rate measurements

To study temperature and salinity responses *Bathycoccus* clades BI (RCC4222), BII (RCC715), and BIII (UST710) were acclimated to specified conditions for 2 weeks. They were then cultured in triplicate under different temperatures (5, 10, 15, 20, 25, and 30°C) in a L1 medium with a salinity of 30‰, or in L1 medium with different salinities (5, 10, 15, 20, 25, 30, 35, 40‰) at a constant temperature of 20°C. Cell concentrations were daily measured with a CLARIOstar Plus microplate reader at 480 nm excitation and 680 nm emission. Growth rates (μ; d^−1^) of the exponential growth phase were calculated according to the equation:


$$ \mu =\frac{\mathit{\ln}\left({N}_t\right)-\mathit{\ln}\left({N}_0\right)}{t} $$

where N_t_ is the cell concentration at time t, N_0_ is the initial cell concentration, t is the duration of time, and μ is the grow rate.

### Electron microscopy

The fresh algal pellet of *Bathycoccus* strain UST710 was collected and fixed with 2.5% glutaraldehyde, rinsed with 0.1 M sodium cacodylate buffer, and post-fixed with 1% osmium tetroxide. The samples dehydrated through a graded ethanol series and embedded with EMbed-812 resin (EMS, USA). Ultrathin sections of the embedded samples were cut using a Leica EM UC7 Ultramicrotome and stained with uranyl acetate and lead citrate. The sections were examined using a Hitachi HT7700 Transmission Electron Microscope.

### Comparison of nutrient metabolism gene content

Metabolic gene content was compared across 21 eukaryotic picophytoplankton genomes (Table S7), including *Bathycoccus* clades (three genomes for each clade, totaling *n* = 12), *Ostreococcus* (*n* = 4), *Micromonas* (*n* = 2), and three typically oligotrophic species (*Chloropicon primus*, *Pycnococcus provasolii*, and *Pelagomonas calceolata*). Gene annotation was performed using BLASTP or HMMER with an e-value of 10^−10^ against several manually curated databases, including NCycDB [35], PCycDB [36], and FeGenie [37], each targeting nutrient metabolism for nitrogen, phosphorus, and iron, respectively. Metabolic gene annotations for Vitamin B_12_, B_1_, and B_7_ were queried against published sequences and KEGG database.

### Analysis of divergence history and gene family evolution

To estimate the divergence time of different *Bathycoccus* clades, analysis was performed on the protein sequences of the 37 *Bathycoccus* genomes, along with reference protein sequences from a number of species in the green lineage (Viridiplantae), which include groups of Prasinophytes, core chlorophytes, Charophytes, and land plants. These sequences were retrieved from public databases (Table S7). An ML tree for the green lineage was constructed using single-copy orthologous genes identified by OrthoFinder v.2.5.5 [31]. Divergence time was estimated using MCMCTree within the PAML v.4.8 [38], using the autocorrelated relaxed clock model. Five calibration points were applied to constrain the age of the nodes (Table S12). The congruence of the results was verified using Tracer v.1.7.1 [39]. Time-calibrated trees were visualized with tvBOT [32]. The expansion and contraction of gene families were inferred by CAFE5 v.5.1.0 [40], with the settings of “-c 20 -l 0.01 -p -k 2”. Significant expanded and contracted gene families (*P*-value<0.05) were analysed for Gene Ontology (GO) enrichment using the OmicShare pipeline (https://www.omicshare.com/tools). Results were visualized with semantic similarity scatterplots in GO-Figure (https://gitlab.com/evogenlab/GO-Figure).

### Analysis of C2H2 zinc finger (C2H2-ZF) and ankyrin repeat (ANK) protein families

To investigate the roles of C2H2-ZF and ANK protein families, candidate proteins from 37 *Bathycoccus* genomes and various other eukaryotic phytoplankton and land plants (Table S13) were identified using hidden Markov models profiles for C2H2-ZFs and ANKs. HMMER was employed with an e-value threshold of 10^−5^ to search protein sequences across these species. Identified proteins were further verified through PROSITE (https://prosite.expasy.org/) and SMART (http://smart.embl-heidelberg.de/), to remove the sequences lacking C2H2-ZF or ANK domains. The proportion of C2H2-ZF or ANK genes in the genome of each species was calculated (Table S13).

## Results and discussion

### Uncovering hidden diversity in *Bathycoccus*

**Figure 1:**
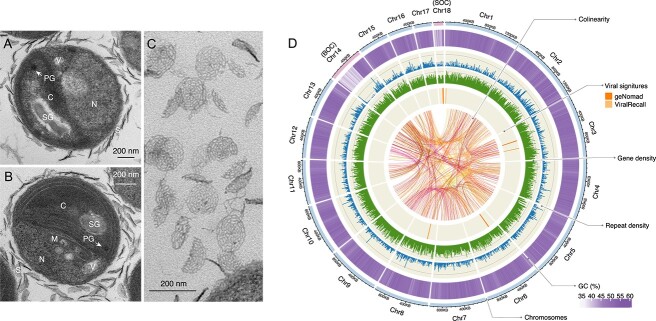
**Morphologic and genomic characteristics of the *Bathycoccus* sp. UST710.** (**A, B**) Transmission electron microscopy (TEM) images of *Bathycoccus* sp. UST710 cells revealing the nucleus (N), single chloroplast (C), mitochondrion (M), vesicles (V), starch grain (SG), plastoglobuli (PG), and scales (S) covering the cell surface. Scale bars: 200 nm. (**C**) TEM image displaying a detailed view of the scales. Scale bars: 200 nm. (**D**) Physical map of the genome highlighting the key features of this isolate. The outermost track illustrates the size of 18 chromosomes, labeled Chr1-18 in descending order of size, with two outlier chromosomes—the BOC and the SOC—labeled and highlighted. Proceeding inward, four tracks represent the distribution of GC content (5-kb sliding windows), repeat element density (10-kb sliding windows), gene density (10-kb sliding windows), and predicted viral regions identified by geNomad and ViralRecall. Syntenic gene blocks, identified by MCScanX, are connected by links at the center.

We successfully isolated a collection of 28 *Bathycoccus* strains from the coastal waters of the northern South China Sea (NSCS) during 2021–2022 (Table S1). These newly isolated strains share high ultrastructural similarities with the well-characterized clades BI and BII [21, 41], with their cell surfaces covered by external scales arranged in eight projections stemming from a central hub (Fig. 1A–C). Meanwhile, a comparison of the widely used V4 region of 18S rRNA gene sequences reveals no noticeable dissimilarities. Instead, phylogenetic analysis based on the ITS1-5.8S-ITS2 region clearly demonstrates that the NSCS strains form a distinct clade, which we propose to designate as BIII (Fig. S1).

To gain genomic insights into this cryptic clade, we meticulously selected the highly purified strain UST710 for whole-genome sequencing. The de novo assembly yielded a streamlined yet highly complete genome (BUSCO completeness: 97%) with a size of 15.34 Mb, encompassing 18 chromosomes, each featuring telomeric repeats (5’-CCCTAAA-3′) at both ends (Fig. 1D, Table S2). The genome contains 7865 predicted genes, with an average gene density of 0.51 genes per kilobase. Only a small portion of the genome (0.7 Mb) was identified as repetitive elements (Table S3). The overall GC content of the genome is 48.48%, similar to the BI and BII genomes. We identified two distinct “outlier chromosomes” with a lower GC content (Fig. 1D), a trait shared among Mamiellophyceae genomes [42].

**Figure 2:**
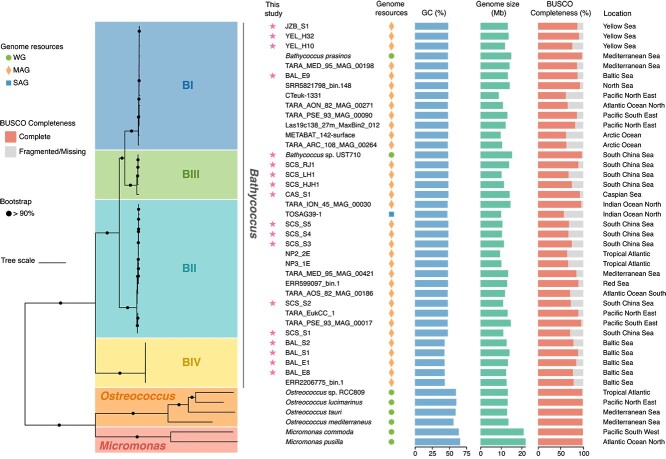
**Phylogeny and genome comparison of four *Bathycoccus* clades, BI, BII, BIII, and BIV.** From left to right: (i) Phylogenomic tree depicting the relationships among 37 qualified genomes of *Bathycoccus* and other Mamiellophyceae members (*Micromonas* and *Ostreococcus*). The tree scale is 0.2. The tree was constructed using the concatenated sequence alignment of single-copy orthologs using the Q.pfam+F + I + R5 model. Taxonomy of the genomes is indicated. Bootstrap support values above 90% are denoted by black dots at the nodes. The scale bar represents branch length; (ii) names of the genomes with new genomes generated from this study marked by stars on the left. Different shapes on the right indicate the types of genome resources (WG for whole genome of the strain; MAG for metagenome-assembled genome; SAG for single-amplified genome); (iii) average GC content; (iv) genome size; (v) genome completeness based on BUSCO; (vi) geographic locations where the genome was recovered. Each qualified genome has a contamination level of <2% and a completeness level of over 50%.

To elucidate the global genomic diversity of *Bathycoccus*, we performed binning on published metagenomic data from diverse marine environments, resulting in 17 novel, high-quality MAGs of *Bathycoccus* (Table S5). Together with the published genomic resources and our novel *Bathycoccus* sp. UST710 genome assembly, we constructed a phylogenomic tree incorporating all 37 *Bathycoccus* genomes, which unveiled the presence of a fourth distinct clade, designated as BIV, alongside clades BI, BII, and BIII (Fig. 2). The BIV clade consists solely of MAGs from the Baltic region, and currently lacks culturable representatives. Further investigations indicated that an uncultured *Bathycoccus* rRNA gene sequence from the Russian Arctic Seas [43] fall within the BIV clade (Methods S1). This finding supports the BIV clade as a distinct and independent lineage within the *Bathycoccus* genus, as elucidated through comprehensive analysis of phylogeny and ITS secondary structure (Fig. S1). The BIV genomes exhibit a lower GC content of ~43% and occupy a basal position in the *Bathycoccus* phylogenetic tree, suggesting that they represent an early-diverged lineage (Fig. 2). Additionally, a pairwise comparison of ANI and AAI across different *Bathycoccus* clades revealed clear interspecific differences. Inter-clade comparisons showed lower similarity (ANI: 76.0–86.2%, AAI: 65.7–84.5%), whereas intra-clade comparisons exhibited high similarity (ANI > 95.88%, AAI > 94.06%) (Fig. S3). This clear separation in both ANI and AAI values between inter-clade and intra-clade comparisons strongly supports the classification of these clades as separate species, aligning with emerging standards in eukaryotic genomics [27, 44, 45].

Our analysis revealed the presence of introns inserted within the 18S rRNA gene regions across all *Bathycoccus* clades, contributing to significant variability among the clades (Fig. S2). These introns, commonly found in eukaryotic rRNA gene sequences, require careful consideration when interpreting diversity [46]. The presence of these introns was not universal in all *Bathycoccus* sequences and absent in other Mamiellophyceae species. Moreover, introns were detected within the 28S rRNA gene regions in two *Bathycoccus* sequences. The presence of rRNA introns and ITS region variability highlights the need for higher resolution approaches, such as long-read amplicon sequencing [47], to investigate their diversity and evolutionary history. Besides, we identified EVEs in the small outlier chromosome (SOC) and four normal chromosomes in the *Bathycoccus* sp. UST710 genome (Table S4a,b). Further investigation revealed the presence of these EVEs across genomes from all *Bathycoccus* clades, with at least 20 distinct types identified (Table S4c), some being clade specific*.* This finding warrants further exploration of the interactions and potential horizontal gene transfer between *Bathycoccus* clades and viruses.

We acknowledge additional genomic diversity within *Bathycoccus* clades likely exists, currently undetected due to limitations in genome recovery from available samples and insufficient exploration of diverse marine environments. Future efforts should integrate metagenomics with Hi-C and long-read sequencing techniques [48, 49] to acquire unexplored *Bathycocus* genomes, as well as larger and more complex genomes from diverse eukaryotic lineages, enabling a more comprehensive exploration of their genetic makeup.

### Distinct ecological niches of *Bathycoccus* clades worldwide

**Figure 3:**
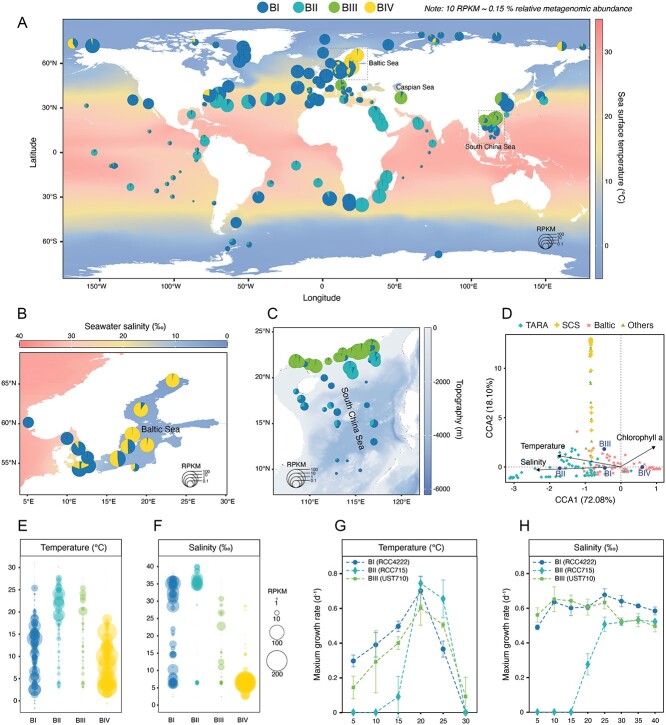
**Global biogeography of four *Bathycoccus* clades and their adaptation to temperature and salinity.** (**A–C**) Distribution of *Bathycoccus* clades BI, BII, BIII, and BIV in the surface water of (**A**) global ocean, (**B**) the Baltic Sea, and (**C**) the SCS, as inferred from metagenomic read recruitment to reference genomes. The size of pie chart represents the relative abundance of all *Bathycoccus* in metagenomic samples, normalized as RPKM (reads per kilobase per million mapped reads). Each pie chart is divided into four sectors, corresponding to the proportion of each clade. The background gradients indicate (**A**) sea surface temperature, (**B**) seawater salinity, and (**C**) topography, respectively. (**D**) CCA illustrating the association between environmental parameters and the abundance of different *Bathycoccus* clades. Data from multiple published studies were included in the analysis, including TARA (Tara Oceans expedition), Baltic (Baltic Sea), SCS, and others (Yellow Sea, Caspian Sea, Chesapeake and Delaware Bay). Only parameters with a significant *P* value (*P* < 0.01) are shown. (**E, F**) Bubble plots illustrate the range of values for two environmental parameters, temperature (**E**) and salinity (**F**) for different *Bathycoccus* clades. The bubble size represents the genome abundance (normalized as RPKM). (**G, H**) Maximum growth rates measured in the laboratory under different temperature (**G**) and salinity (**H**) conditions, revealing specific growth responses to temperature and salinity for culturable *Bathycoccus* clades, BI (strain RCC4222), BII (strain RCC715), and BIII (strain UST710).

To investigate the global distribution and ecological niches of *Bathycoccus* clades, we scrutinized 457 publicly available metagenomic samples from a broad range of marine environments, specifically focusing on the photic zones of the oceans (Table S9). Through metagenomic read mapping to the representative genome of each clade, we quantified their relative abundance worldwide. *Bathycoccus* was found across major ocean biogeographical provinces, consistent with previous findings [19, 20] (Fig. 3A and S5). These algae displayed a preference for coastal waters over oligotrophic waters, and were scarce in high-nutrient, low-chlorophyll regions (HNLC), including the Southern Ocean, Equatorial Pacific, and Subarctic Pacific. Among the 143 stations with abundant *Bathycoccus* (defined as total *Bathycoccus* RPKM >1), a single clade dominated in 86.7% of these stations, accounting for >90% of *Bathycoccus* abundance. Transitional zones, exemplified by the vicinity of Gulf Stream and the confluence of the North Sea with the Baltic Sea, were exceptional in featuring two co-dominant clades, whereas the coexistence of three or more clades was a rarity, indicating distinct ecological preferences among the clades.

We integrated genomic abundance data with measured environmental parameters to identify the major drivers of their global biogeographic patterns (Fig. 3A–F). Canonical Correspondence Analysis showed clearly differentiated ecological niches for each *Bathycoccus* clade, pinpointing temperature and salinity as pivotal factors in clade distribution and the delineation of the distinct ecotypes (Fig. 3D). Clade BI emerged as an ecological generalist, thriving across a broad thermal range (0–25°C) from subtropical to polar waters, and capable of tolerating a broad salinity spectrum (6–36‰). In contrast, clade BII was characterized as a specialist, with narrow thermal (18–28°C) and salinity ranges (34–40‰), preferring warmer and saltier waters, such as the Indian Ocean and Red Sea. Clade BIII was more abundant in coastal environments, including nearshore and estuarine waters in the SCS, Yellow Sea, and Adriatic Sea. Intriguingly, clade BIII was also prevalent in the Caspian Sea (Fig. 3A), which was historically connected to the world ocean as part of the ancient Paratethys Sea. Despite becoming geographically isolated ~14 million years ago [50], BIII has persisted in this unique habitat and maintains a high genetic similarity (ANI > 96%) with BIII populations in other waters. Clade BIV primarily inhabited cooler, less saline waters (1–18°C, 2–10‰), such as the Baltic Sea, Arctic marginal seas, and regions experiencing temperate winters with low salinity, such as Chesapeake Bay.

To further unravel the biogeographic patterns of *Bathycoccus* clades within regional waters, we assessed their distribution along environmental gradients in the SCS and the Baltic Sea (Fig. 3B, C). In the SCS, there was a notable transition from clade BIII coastal dominance to clade BII offshore predominance, coinciding with decreasing nutrient availability from the coast to the open sea [51]. Although the SCS basin presented a lower overall presence of *Bathycoccus*, a dominance by clade BI was detected. This segregation of *Bathycoccus* clades suggests their adaptations to varying nutrient availability. In the Baltic Sea’s brackish water, characterized by pronounced salinity gradients [52], there was a clear transition from clade BIV in the north to clade BI in the southwest (Figs 3B and S4), suggesting their differentiated salinity preferences. Though clade BIV remains uncultured, our metagenomic analyses in biogeographic surveys have revealed the niche preferences of different clades. This information can direct efforts to isolate clade BIV from specific environments, such as the Baltic Sea.

To complement our metagenomic survey, we conducted growth rate experiments on representative strains of clade BI, BII, and BIII, evaluating their physiological responses across various temperatures and salinities (Fig. 3G, H). These experiments reinforced the distinct physiological adaptations of these clades, mirroring the ecological preferences observed in their natural habitats. For example, clade BI, which thrives in cold waters, exhibited the fastest growth in 5°C among the three clades (*P* value <0.05, t test). Clade BII, inhabiting warmer and saltier waters, demonstrated a coherent preference under laboratory conditions. Conversely, clade BIII displayed wider tolerance ranges for temperature and salinity, suggesting that additional factors, such as nutrient availability, are also crucial in their niche adaptation.

### Genomic basis for nutrient adaptation

Mamiellophyceae generally prefer coastal waters, yet certain clades such as *Bathycoccus* Clade BII and *Micromonas commoda* also thrive in the open ocean [19]. Conversely, certain eukaryotic picophytoplankton species, such as *C. primus*, *P. calceolata, and P. provasolii*, dominant exclusively in oligotrophic waters [18, 53, 54]. We analysed the nutrient metabolism gene content among these taxa, which are comparable in cell and genome size, to elucidate their adaptive potential to specific nutrient regimes.

**Figure 4:**
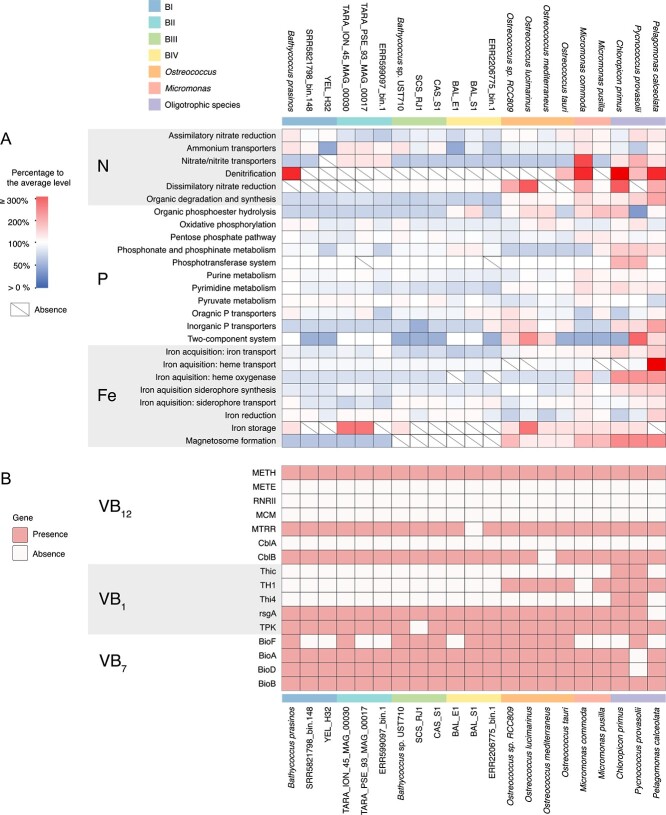
**Comparison of nutrient metabolism gene content among eukaryotic picophytoplankton.** The selected 21 genomes of eukaryotic picophytoplankton include four *Bathycoccus* clades, *Micromonas*, *Ostreococcus*, and three oligotrophic species. (**A**) The heatmap depicts differences in gene content involved in nitrogen (N), phosphorus (P), iron (Fe) metabolism among the eukaryotic picophytoplankton. The heatmap gradient indicates whether the gene copy number for a specific process is overrepresented, equally represented, or underrepresented compared with the average level of the selected genomes. Boxes with a diagonal line indicate the absence of genes associated with a particular process. (**B**) The binary heatmap displays the presence (red) or absence (white) of genes encoding vitamin B_12_ (VB_12_)-dependent enzymes (METH, RNRII, MCM), VB_12_-independent enzyme (METE), and their accessory proteins (MTRR, CblA, CblB), as well as proteins involved in biosynthesis of vitamin B_1_ (VB_1_) and vitamin B_7_ (VB_7_).

Nitrogen (N), phosphorus (P), and iron (Fe) are key nutrients that influence the distribution and productivity of marine primary producers [55]. Our comparative genomic analysis (Fig. 4A, Table S10) reveals that species typically found in oligotrophic waters often possess more genes for nitrate/nitrite transporters (NRT2 type) and inorganic phosphate transporters (PstS, pho4, PiT). In contrast, these genes are scarce in *Bathycoccus* genomes. Additionally, genes responsible for sensing and responding to N or P deficiency, including nitrate/nitrite sensor (NIT), alkaline phosphatase (phoA,X), and phosphate starvation-inducible ATPase (phoH), are entirely missing in this genus (Fig. 4A, Table S10). The absence of these genes, along with the paucity of genes for iron acquisition in *Bathycoccus*, underscores its evolutionary adaptation to nutrient-rich coastal environments. Nonetheless, *Bathycoccus* clade BII is an exception with distinctive genomic features, such as the presence of an additional NarK/NasA type nitrate/nitrite transporter gene, and a surplus of ferritin genes, crucial for managing iron storage and homeostasis in phytoplankton [56]. This gene enrichment may provide clade BII with an adaptive advantage for survival in nutrient-depleted conditions, aligning with their distribution in oligotrophic marine environments.

Eukaryotic phytoplankton commonly exhibit auxotrophy for certain B vitamins essential for key metabolic processes, including cobalamin (B_12_), thiamine (B_1_), and biotin (B_7_). These vitamins must be acquired from their surroundings [57]. Our investigation found that all *Bathycoccus* clades possess the gene encoding B_12_-dependent methionine synthase (METH), yet they lack the gene for the alternative B_12_-independent isoform of this enzyme (METE), suggesting their reliance on external sources of B_12_ for growth (Fig. 4B). Furthermore, the absence of genes responsible for B_1_ biosynthesis, namely TH1, ThiC, and Thi4, in all *Bathycoccus* clades, suggesting their B_1_-auxotrophy (Fig. 4B). Conversely, oligotrophic species, including *C. primus* and *P. provasoli*, possess all these genes, suggesting their capability to synthesize B_1_. Nevertheless, all *Bathycoccus* clades contain a complete B_7_ biosynthesis pathway, indicating self-sufficiency in vitamin B_7_ and eliminating the need for external B_7_ sources.

### Climate-driven speciation and gene family evolution in *Bathycoccus*

**Figure 5:**
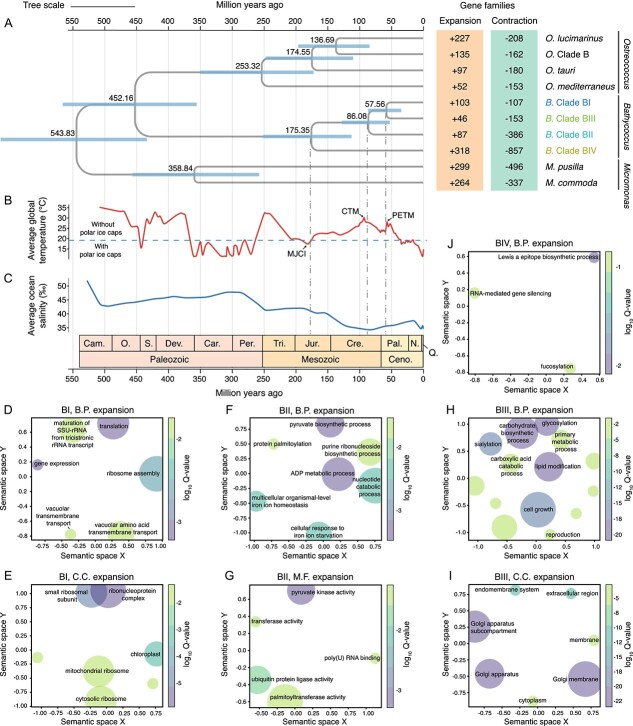
**Divergence history and gene family evolution within clades in *Bathycoccus*.** (**A**) Left: a time-calibrated phylogenetic tree illustrating the divergence time of clades in *Bathycoccus*. The tree scale is 1. Divergence times (million years ago, Ma) were inferred using MCMCTree under an autocorrelated relaxed clock model. The mean and the 95% highest posterior density interval of the ages are shown above each node and represented by horizontal bars, respectively. The geologic time scale is based on the Geological Society of America. Abbreviations of geologic period: Cam., Cambrian; O., Ordovician; S., Silurian; Dev., Devonian; Car., Carboniferous; Per., Permian; Tri., Triassic; Jur., Jurassic; Cre., Cretaceous; Pal., Paleogene; N., Neogene; Q., Quaternary; Ceno., Cenozoic. Only the Mamiellophyceae section of the tree is shown (the full time-calibrated tree of the green lineage is provided in Fig. S6); right: evolutionary analyses of gene family expansions and contractions for each species or clade in Mamiellophyceae, with a focus on *Bathycoccus*. (**B**) Global average surface temperature over the past 500 million years (data source: Smithsonian National Museum of Natural History). Periods with temperature below (above) the horizontal dotted line indicate the presence or absence of persistent polar ice caps. The divergence times of *Bathycoccus* clades are approximated to coincide with several climatic events, including MJCI (174 to 164 Ma), CTM (94 to 82 Ma), and PETM (Paleocene-Eocene Thermal Maximum, 56 Ma). (**C**) Average ocean salinity over the past 500 million years (data source [61]). (**D–J**) Semantic similarity scatterplots of GO term enrichment (M.F., molecular function; B.P., biological process; C.C., cellular component) of the expanded gene families within the four *Bathycoccus* clades (BI, BII, BIII, and BIV). The plots were generated using the Python package GO-Figure, which clusters similar GO terms and selects one as representative. Circle sizes are scaled based on the number of terms they represent. Circles representing terms that are most similar in semantic space on axes *X* and *Y* are placed closest to each other. The gradient of each circle indicates the significance (log_10_ Q-value) of the corresponding GO term, with only the 10 most significant terms displayed. Full lists of terms and their groupings are available in Table S11.

To estimate time of speciation within *Bathycoccus* genus, we constructed a time-calibrated phylogenetic tree encompassing green algae and land plants ( Figs 5A and S6). Our analysis reveals a compelling association between the divergence of *Bathycoccus* clades and major paleoclimatic events, which correspond to their respective thermal niches (Fig. 5B, C). The earliest diverged clade, BIV, appears to have originated ~175.35 million years ago (Ma), coinciding with the Middle Jurassic Cool Interval (MJCI, 174 to 164 Ma). This period experienced an abrupt drop in seawater temperature [58], which may have led to the preference for cold-water environments observed in BIV today. Clade BII seems to have emerged ~86.08 Ma during the Cretaceous Thermal Maximum (CTM) (94 to 82 Ma), a period of prolonged hot greenhouse climate conditions [59] that likely shaped BII into a warm-adapted specialist. Clades BI and BIII diverged ~57.56 Ma, aligning with the onset of the Eocene epoch (56–34 Ma). This era was characterized by a transition from a hot strike of the Paleocene–Eocene Thermal Maximum (56 Ma) toward a coolhouse that culminated in the late Eocene glaciation [60]. The ability of BI and BIII to withstand such variable temperatures may explain their present-day high thermal tolerance. These insights suggest the influential role of environmental factors, particularly temperature, in steering the speciation and niche differentiation within the *Bathycoccus* genus.

GO enrichment analysis of significantly expanded and contracted gene families in *Bathycoccus* clades reveals distinct functional traits tailored to their specific environmental challenges. The generalist clade BI shows expansion of gene families associated with ribosome assembly and translation (Fig. 5D, E). These traits may provide BI with selective advantages by allowing swift adaptation to fluctuating environments through an increased protein synthesis capacity. In the warm-adapted clade BII, expanded gene families are enriched in GO terms associated with cellular response to iron starvation, as well as, ubiquitination, a key process for cellular recovery following heat shock [62]. This suggests an adaptation to the warm, nutrient-limited environments that BII occupies (Fig. 5F, G). Moreover, the enrichment of expanded genes involved in pyruvate and adenosine diphosphate (ADP) metabolic processes indicates an enhanced ability to generate adenosine triphosphate (ATP) through glycolysis, potentially energizing BII to trigger ATP-dependent stress responses. Clade III shows an expansion of genes linked to the Golgi apparatus and its related functions, including sialylation, glycosylation, and lipid modification (Fig. 5H, I). These biochemical processes likely promote the secretion of various molecules, such as signaling factors, which may confer adaptive benefits to clade BIII for interacting with other microbes in coastal ecosystems. In contrast to clade BII, the cold-adapted clade BIV shows a reduction in genes related to ubiquitination, signaling a decreased reliance on the cellular repair mechanisms critical in warmer conditions and suggests that clade BIV may employ alternative strategies for protein regulation to manage cold stress (Table S11). Moreover, clade BIV shows enrichment for only a few GO terms, implying its adaptations may hinge on regulatory modulation or the versatile use of existing genes (Fig. 5J). These dynamic shifts in gene family composition within *Bathycoccus* highlight the functional adaptations that underpin the resilience and ecological success of these diverse clades.

### Potential role of C2H2 zinc finger and ankyrin repeat-containing proteins in cold adaptation for eukaryotic phytoplankton

**Figure 6:**
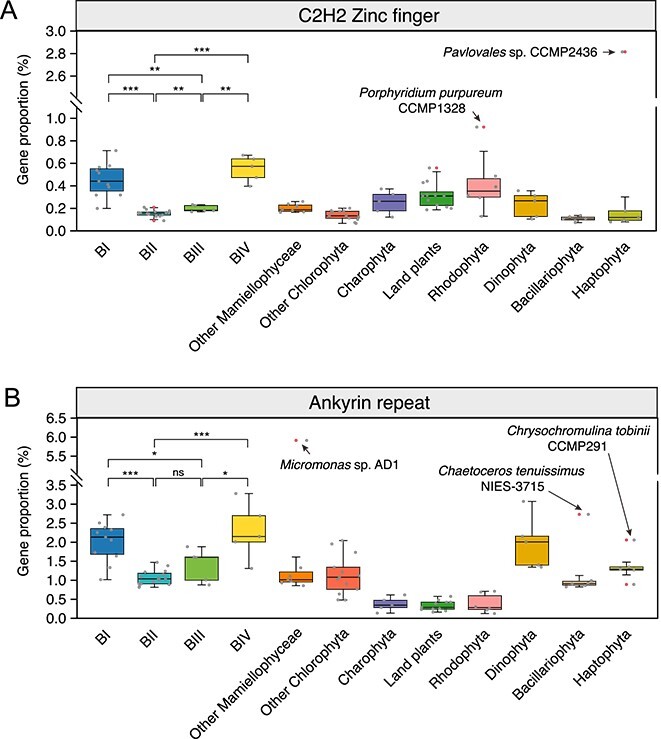
**Comparison of gene proportion of C2H2 zinc finger and ankyrin repeat protein families among genomes of eukaryotic phytoplankton and land plants.** (**A, B**) The box plots show the proportions of C2H2 zinc finger (**A**) and ankyrin repeat (**B**) gene families in the genomes of the four *Bathycoccus* clades, other eukaryotic phytoplankton groups and land plants. For both box plots, the gene proportions in each genome are shown as standard dots, whereas distnct dots represent outlier values. Five eukaryotic phytoplankton with exceptionally high gene proportions (outlier values) are labeled. The gene proportion for both protein families were compared between different *Bathycoccus* clades, an asterisk (^*^) for a *P*-value <0.05, double asterisks (^*^^*^) for a *P*-value <0.01, triple asterisks (^*^^*^^*^) for a *P*-value <0.001, and “ns” for no significant difference (Mann–Whitney U test).

The C2H2-type zinc finger (C2H2-ZF) proteins are one of the largest transcription factor families [63], and ankyrin repeat (ANK) domains are widespread motifs that mediate protein–protein interactions [64]. Both are recognized for their crucial roles in abiotic stress resistance in land plants [63, 65]. Research on the distribution and functions of these proteins in diverse eukaryotic phytoplankton remains limited, as studies have primarily focused on a few species, including *B. prasinos* from Clade BI [41]. Here, we examined the prevalence of C2H2-ZF and ANK gene families within the genomes of four *Bathycoccus* clades and multiple eukaryotic phytoplankton phyla. Our findings show that clade BII, a warm specialist, has the lowest average proportion of both gene families (Fig. 6). In contrast, clades BI and BIV, which thrive in colder waters, display higher proportions of C2H2-ZF and ANK genes compared with *Bathycoccus* clades BII and BIII, as well as most analysed eukaryotic phytoplankton (*P* < 0.05, Mann–Whitney U test). Yet, five genomes, including those of *Pavlovales* sp. CCMP2436 and *Micromonas* sp. AD1—both inhabit polar waters [14, 66]—exhibit pronounced enrichment of these gene families (Fig. 6). The observed expansion of C2H2-ZF and ANK genes in cold-adapted species suggests their potential roles in the cold tolerance. This hypothesis aligns with observations of the adaptative expansion and expression of zinc finger and other zinc-binding protein families in polar phytoplankton [67, 68]. These findings, in conjunction with our results, suggest a potential role for various zinc finger proteins in the cold adaptation mechanisms. The remaining three species, though non-polar, are well-adapted to a broad range of environmental conditions, such as varying salinity levels. This adaptability hints at the potential roles of C2H2-ZF and ANK protein families in managing other environmental stress. Future research should investigate the multi-omics profiles of C2H2-ZF and ANK proteins under various stressors to uncover their roles in stress resistance, crucial for understanding phytoplankton adaptation to changing oceans.

## Conclusions

Eukaryotic phytoplankton display an immense diversity and are extensively distributed across the global ocean [5]. Our study focused on the cosmopolitan picoeukaryotic phytoplankton *Bathycoccus* and revealed hidden diversity within this genus through the analysis of 37 *Bathycoccus* genomes. Our work showcases the potential of culture-independent metagenomic methods to obtain high-quality eukaryotic genomes, overcoming the challenges associated with cultivation and genome assembly in eukaryotes. Moving beyond the earlier view of *Bathycoccus* as a single species, we have identified four distinct clades, with each possessing unique genomic traits, ranging from differences in genomic GC content to distinct gene repertoires. These genome diversifications are intricately connected to niche adaptation and biogeography of each clade, influenced by factors like temperature, salinity, and nutrient availability. A notable discovery in our study is the association between the presence of C2H2 zinc finger and ankyrin repeat genes and a clade’s capacity to thrive in colder waters. Each *Bathycoccus* clade occupies a distinct ecological niche, collectively covering a diverse array of environmental conditions. This diversity underpins the widespread presence of *Bathycoccus* in the global ocean. Similar patterns of genomic diversification, leading to distinct ecotypes within a single “species,” have been observed in other cosmopolitan eukaryotic phytoplankton, such as the green algae *Ostreococcus* and *Micromonas* [13, 69], the coccolithophore *G. huxleyi* [12, 70, 71], and the diatom *Chaetoceros* [72, 73]. Our findings add to the growing body of evidence that microdiversity is common in eukaryotic phytoplankton, suggesting that seemingly single taxonomic units may actually be intricate assemblages of genospecies, reflecting differences in their physiology, niche adaptation, and ecological functions.

Environmental variability and geographic barrier are key factors driving genomic differentiation in marine phytoplankton [74]. Our biogeography and evolutionary analysis reinforce the importance of environmental selection, particularly temperature changes, in the speciation of *Bathycoccus* [21, 75], whereas geographic barriers are more significant in the diversification of other phytoplankton groups such as *Gephyrocapsa* [12] and *Pseudo-nitzschia pungens* [76]. In contrast, the diversification of outlier chromosomes in *Bathycoccus* and other Mamiellophyceae appears to be shaped by horizontal gene transfer, because a substantial proportion of their non-orthologous genes originating from viruses and prokaryotes. This process contributes to the observed hypervariability within these phytoplankton groups [42, 76]. With the ocean warming, the structure of eukaryotic phytoplankton communities undergoes significant transformations [77, 78], which would have profound ecological repercussions due to their roles in marine food webs and biogeochemical cycles. In this context, concerted research efforts are necessary to combine cultivation-dependent and -independent approaches. This integrated approach will enable a deeper understanding of the genomic diversity, adaptive mechanisms, and ecological consequences of *Bathycoccus* and other eukaryotic phytoplankton, thereby unraveling their ecological significance and their responses to ongoing global changes.

## Supplementary Material

Supplementary_figures_and_method_2nd_ISMEsubmission_clean_wrae163

Supplementary_Table_2nd_submission_final_version_wrae163

## Data Availability

The *Bathycoccus* sp. UST710 strain has been deposited at the Roscoff Culture Collection with RCC number of RCC11004. Sequencing reads and the genome assembly for *Bathycocc*us sp. UST710 have been deposited at NCBI GenBank under BioProject accession PRJNA1080260 and BioSample accession SAMN40123937. The study also generated 17 metagenome-assembled genomes (MAGs), which are available in GenBank under BioProject accession PRJNA1080806 and BioSample accession from SAMN40146504 to SAMN40146520. rRNA gene and ITS sequences obtained in this study are available in GenBank, with accession numbers from PP409567 to PP409572. The source of reference genomes, sequences, raw reads analysed in this study can be found in Tables S7–S9, respectively. The information and parameters for bioinformatic tools used in this study can be found in Table S14.
