# Supplementary material for: Hidden genomic diversity drives niche partitioning in a cosmopolitan eukaryotic picophytoplankton
Source: ISME J. 2024 Aug 14;18(1):wrae163. doi: 10.1093/ismejo/wrae163 (PMC11409870; doi:10.1093/ismejo/wrae163)
Supplement: Supplementary_figures_and_method_2nd_ISMEsubmission_clean_wrae163 [file supplementary_figures_and_method_2nd_ismesubmission_clean_wrae163.docx]

Supplementary Figures and Methods for

**Hidden genomic diversity drives niche partitioning in a cosmopolitan eukaryotic picophytoplankton**

**Yangbing Xu^1^, Shara K.K. Leung^1^, Taylor M.W. Li^1^ and Charmaine C.M. Yung^1*^**

^1^ Department of Ocean Science, The Hong Kong University of Science and Technology, Hong Kong SAR

*** Corresponding author:**

Charmaine C.M. Yung
ccmyung@ust.hk

**Supplementary Figures**

**
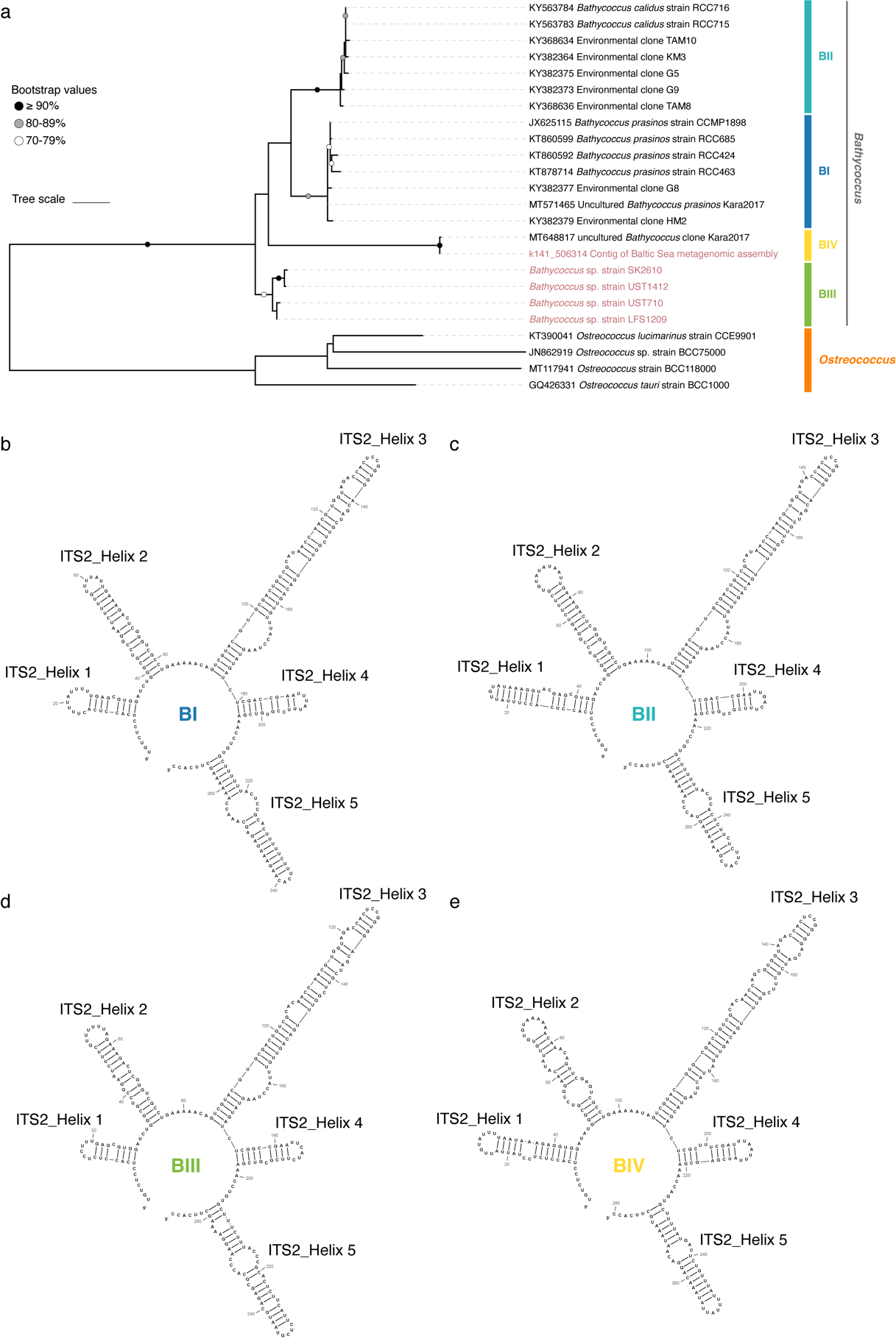
**

**Fig. S1 The internal transcribed spacer (ITS) sequences in *Bathycoccus*. a,** Maximum-likelihood (ML) phylogenetic tree of Bathycoccaceae ITS1-5.8S-ITS2, including the flanking 18S and 28S rRNA gene regions, with a specific focus on *Bathycoccus*. The tree was reconstructed using IQ-TREE under K2P+I+G4 model. Bootstrap values greater than 70% (based on 1000 replicates) are displayed at the nodes using black (> 90%), grey (80-89%), or white (70-79%) circles. *Ostreococcus* species were used as an outgroup. Sequences from this study are highlighted in red, including four sequences from strains and one sequence from a metagenomic assembly. **b-e,** Molecular signatures of four *Bathycoccus* clades revealed by comparing the secondary structures of the ITS2 region. The five major helices are labeled as ITS2_Helix 1 to 5.


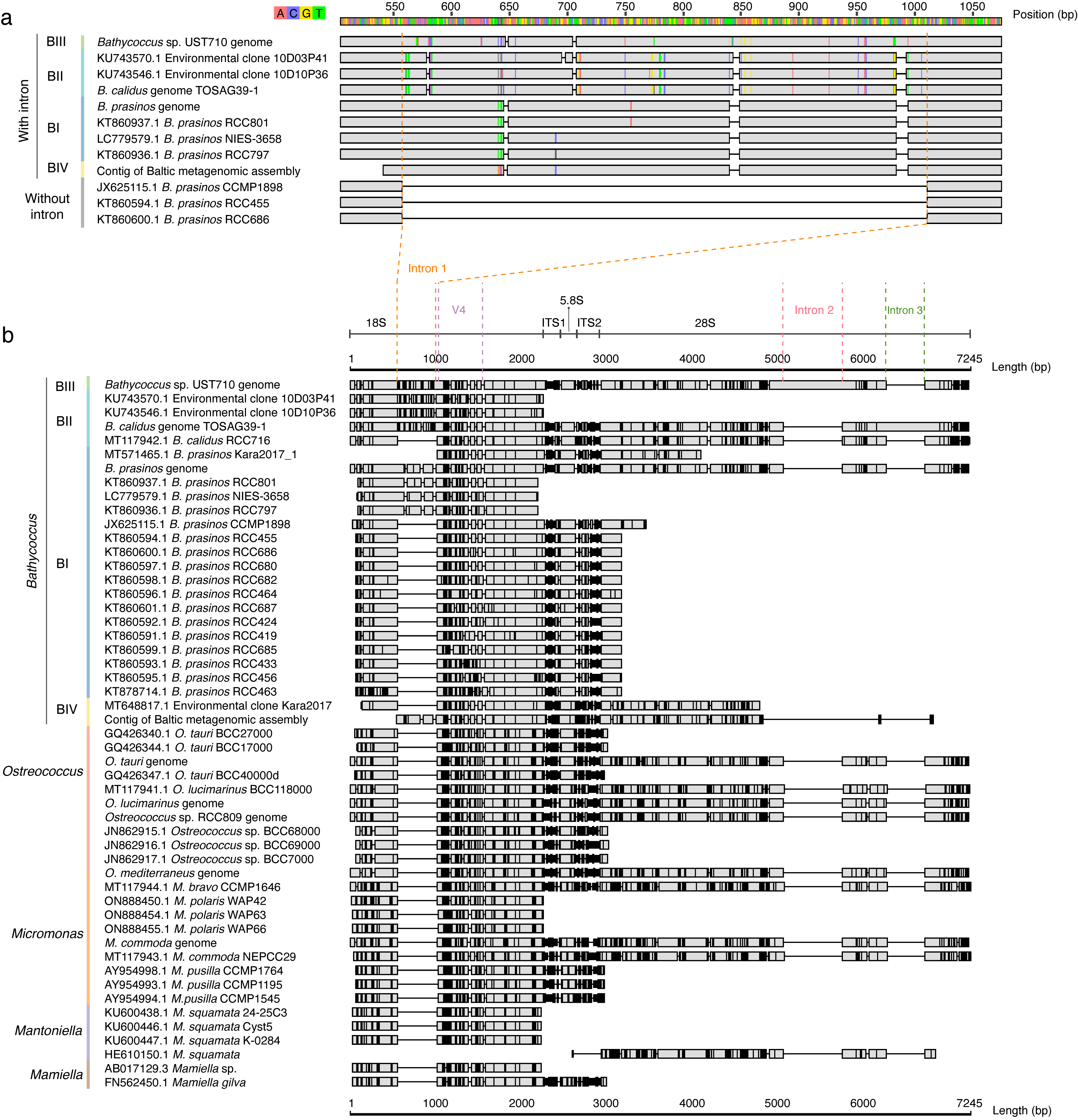


**Fig. S2 Intron insertions in rRNA gene sequences of *Bathycoccus*.** **a,** Detection and alignment of intron in 18S rRNA gene sequences of *Bathycoccus* clades, with a comparison of sequences without intron. The consensus sequence in the alignment indicates the nucleotide composition and its position in 18S rRNA gene sequence. **b,** Comprehensive view of intron regions (intron 1, 2, 3) in full 18S-ITS1-5.8S-ITS2-28S rRNA gene sequences, comparing the intron presence among *Bathycoccus* clade and other genera within the class of Mamiellophyceae. The position of 18S variable region V4, commonly used for studying the diversity and phylogeny of eukaryotes, is also indicated. Consensus regions are depicted using grey color, while nucleotides that disagree among sequences are highlighted in red, purple, yellow, or green in **(a)** or black in **(b)**. Gap regions are indicated as horizontal lines. The alignment was generated using Geneious Prime.


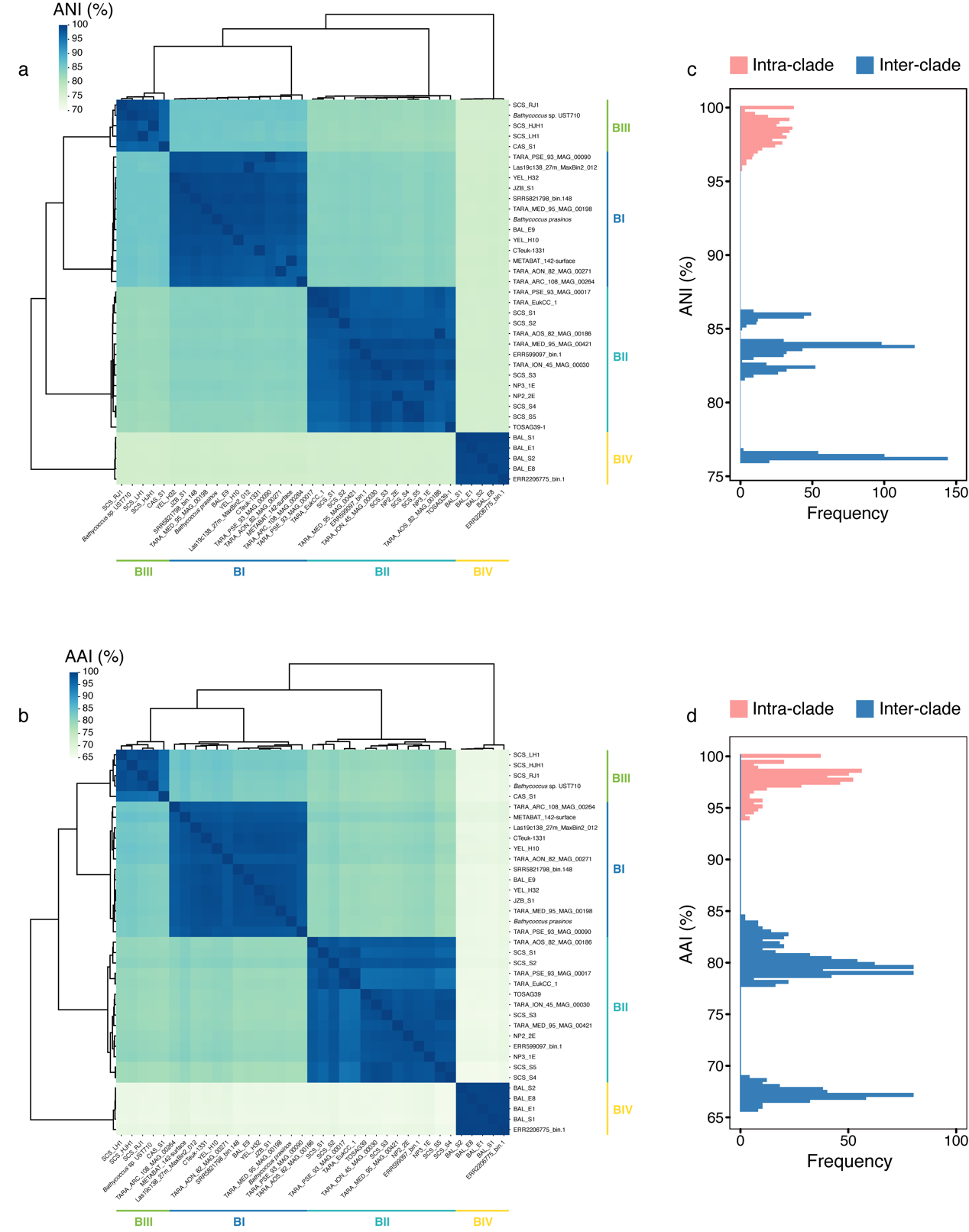


**Fig. S3 Pairwise comparisons of the average nucleotide identity (ANI) and average amino acid identity (AAI) among 37 qualified genomes of *Bathycoccus* revealing four clades.** **a,b,** Heatmaps display color gradient ranging from light green (representing low identity) to dark blue (representing high identity), indicating the ANI **(a)** and AAI **(b)** values between pairs of genomes. Genomes that shared ANI values above 95% **(a)** or AAI values above 94% **(b)** are clustered together and the four clades (BI, BII, BIII, BIV) are labeled with color blocks and texts. **c,d,** Histograms demonstrate the frequency and distribution of of ANI **(c)** and AAI **(d)** values among the 37 *Bathycoccus* genomes. Red color represents intra-clade comparisons, and blue color represents inter-clade comparisons. Each qualified genome has a contamination level less than 2% and a completeness level more than 50%.

**
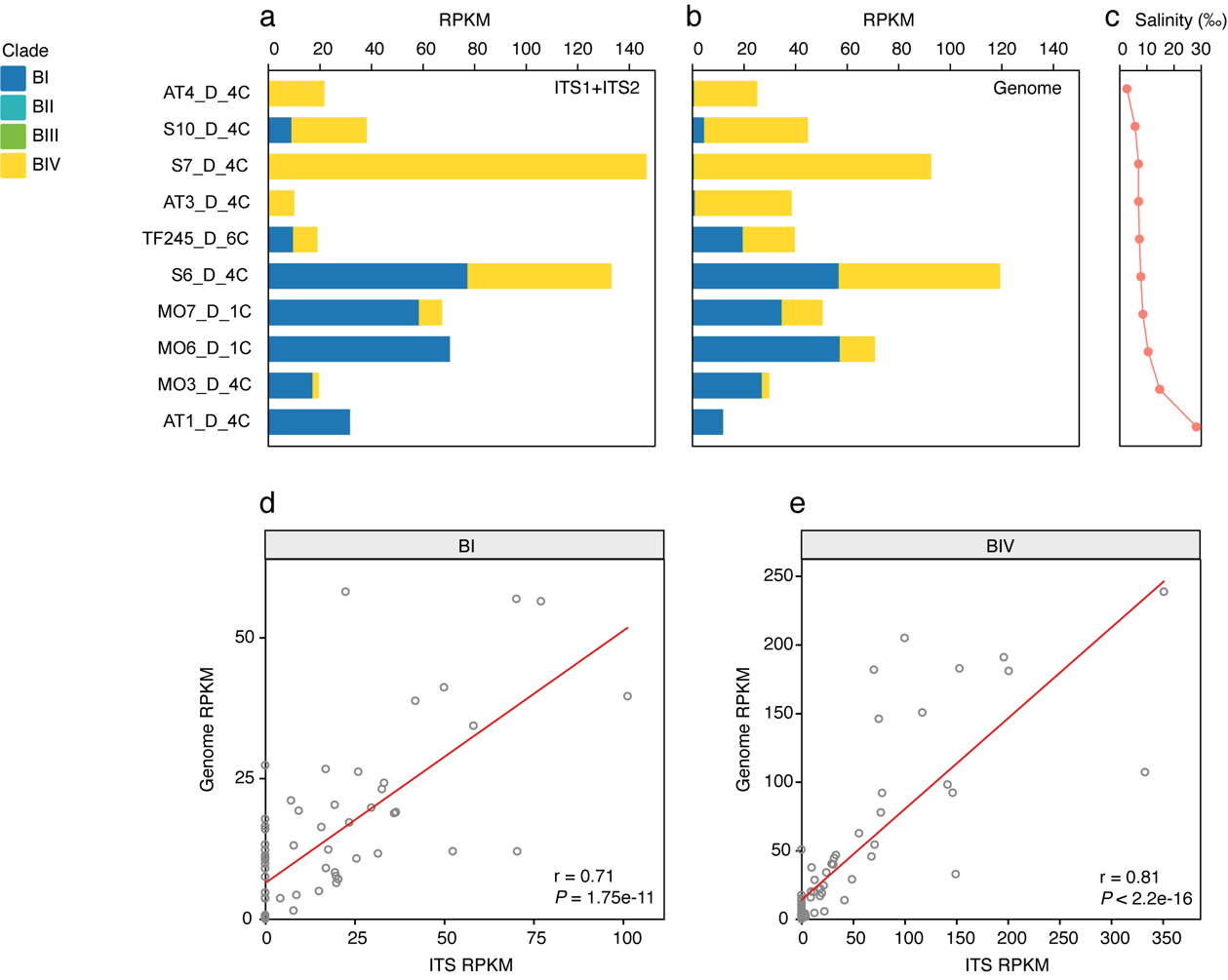
**

**Fig. S4 Evidence demonstrating concordance between the genomes and the ITS sequences in *Bathycoccus* clade BIV.** **a,b,** Stacked histograms displaying the relative abundance of *Bathycoccus* clades in surface water at various locations in the Baltic Sea. The abundance was determined through metagenomic read recruitment to ITS (ITS1+ITS2) regions **(a)** or genomes **(b)**, and the values have been normalized as RPKM (reads per kilobase per million mapped reads). The station locations arranged from inner water to outer water (from top to bottom). **c,** Surface seawater salinity levels across different locations. **d,e,** Scatterplots showing the positive correlation between the read recruitment results for relative abundance using ITS regions (ITS RPKM) and genomes (genome RPKM) in BI (**d**; Pearson’s correlation r = 0.71, p-value = 1.75e^-11^) and BIV (**e**; Pearson’s correlation r = 0.81, p-value < 2.2e^-16^).


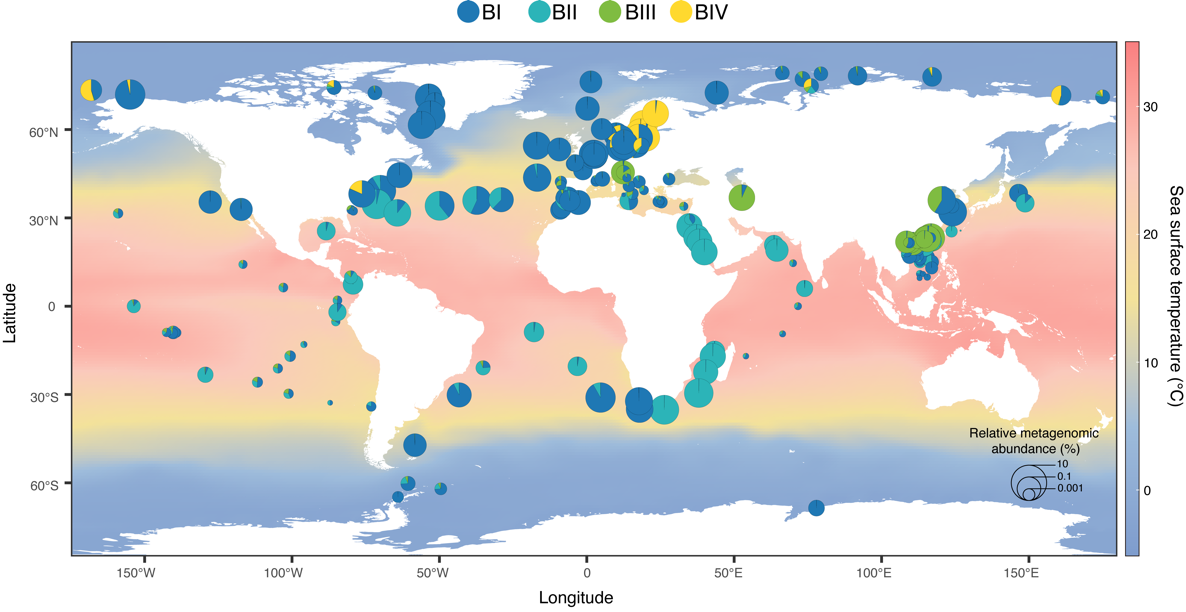


**Fig. S5 Global biogeography of four *Bathycoccus* clades (results without normalization).** Global distribution of *Bathycoccus* clades (BI, BII, BIII, BIV) in surface water was inferred from metagenomic read recruitment to genomes. The size of pie chart represents the relative abundance of all *Bathycoccus* in metagenomic samples, without normalization. Each pie chart is divided into four sectors, representing the proportion of different clades. The background color gradients represent sea surface temperature.


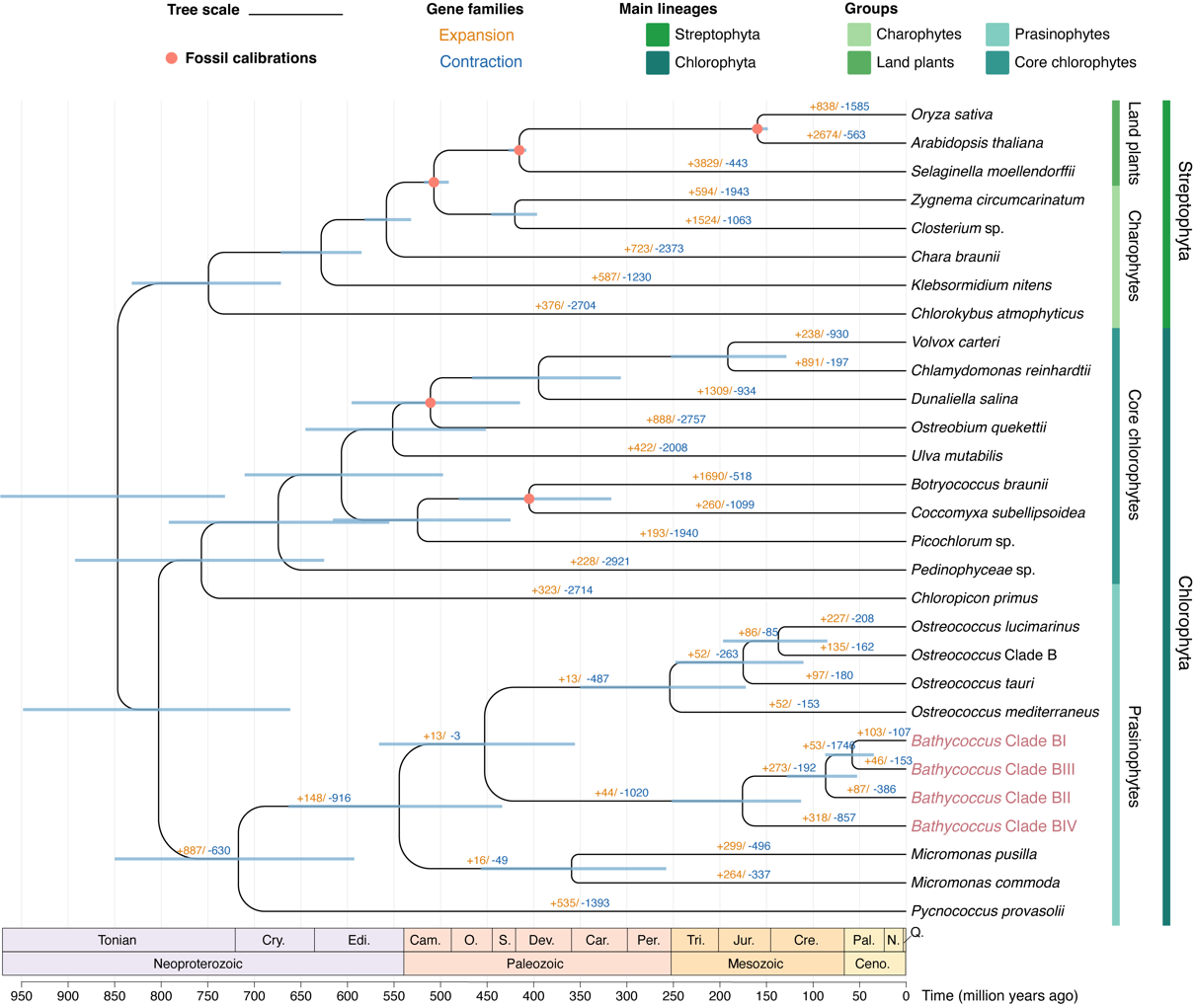


**Fig. S6 Divergence history and gene family evolution for the green lineage (Viridiplantae).** A time-calibrated phylogenetic tree demonstrates the divergence time of various clades within *Bathycoccus*, as well as other species within the green lineage. The tree scale is 1. Their taxonomy is indicated using color blocks and labels on the right. The names of *Bathycoccus* clades are highlighted in red. Divergence times (million years ago, Ma) were inferred using MCMCTree under an autocorrelated relaxed clock model. The 95% highest posterior density (HPD) interval of the age is represented by a blue horizontal bar at each node. Nodes that have been calibrated using fossil evidence are indicated with red circles. The geologic time scale is based on the Geological Society of America. Abbreviations of geologic period: Cry., Cryogenian; Edi., Ediacaran; Cam., Cambrian; O., Ordovician; S., Silurian; Dev., Devonian; Car., Carboniferous; Per., Permian; Tri., Triassic; Jur., Jurassic; Cre., Cretaceous; Pal., Paleogene; N., Neogene; Q., Quaternary; Ceno., Cenozoic. The number of significantly expanded (orange text) and contracted (blue text) gene families is shown above the branches.


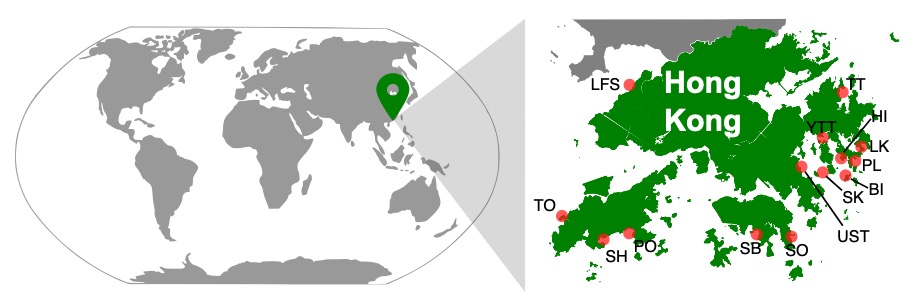


**Fig. S7 Locations for sampling and algal strain isolation in Hong Kong coastal waters.** The red dots indicate the sampling locations, with abbreviations of the location names: LFS, Lau Fau Shan Pier; TO, Tai O; SH, Shui Hau; PO, Pui O; SB, South Bay; SO, Shek O; SK, Sai Kung PM7 station; UST, HKUST Pier; YTT, Yim Tin Tsai; HI, High Island; BI, Bluff Island; PL, Pak Lap; LK, Long Ke; TT, Tai Tan.

**Supplementary Methods**

**Method. S1 Proofs to show that the genomes and the ITS sequences belong to the same *Bathycoccus* clade BIV.**

We obtained 5 metagenome-assembled genomes (MAGs) from the Baltic Sea, and the genome comparison demonstrated that they belong to a novel *Bathycoccus* clade distinct from the other three known clades (Fig. 2, S3). Previous metabarcoding studies targeting on rRNA gene regions have suggested the existence of a novel clade in the Russian Arctic Seas and the Baltic Sea [1, 2]. Interestingly, these findings originated from similar environments characterized by cold and low-salinity waters at high-latitudes, greatly influenced by river input. This similarity suggests a likelihood that the MAGs and the rRNA gene sequences belongs to the same *Bathycoccus* clade, an issue need to be addressed to determine the total number of known clades to date.

The most straightforward approach to answer this question would be to isolate the strains and to sequence their rRNA gene sequences and genomes. However, due to our study being limited to subtropical waters, we faced challenges in isolating strains belonging to this (or these) clade(s). Besides, all the MAGs we obtained lack the rRNA gene regions (or only have very limited part to distinguish different clades), except for the 5S part, despite employing multiple assembly and binning strategies. This common issue is likely caused by differences in copy numbers and tetranucleotide frequencies between rRNA gene sequences and other genomic regions [3]. Consequently, we were unable to find direct evidence through these means.

Therefore, we focused our investigation on publicly available metagenomic datasets from the Baltic Sea [4], where the relative metagenomic abundance of the putative novel clade was exceptionally high. The relative abundance was inferred mapping metagenomic reads from Baltic Sea samples to a representative MAG from the Baltic Sea and normalized as RPKM (reads per kilobase per million mapped reads). We also calculated the relative abundance for the rRNA gene sequence from the Russian Arctic Seas by recruiting reads of the Baltic Sea metagenomic samples to its variable internal transcribed spacer region (ITS1 + ITS2). Comparison of the relative abundance for the MAGs (genome RPKM) and the rRNA gene sequences (ITS RPKM) in surface water across different locations in the Baltic Sea (Fig. S4a-c) demonstrated a consistent trend: the *Bathycoccus* communities shifted from being dominated by the putative novel clade to being dominated by BI clade, as the locations transitioned from low-salinity (2.44‰) inner water to high-salinity (28.05‰) outer water. A similar trend was observed in the *Bathycoccus* communities of the Russian Arctic Seas, as identified through metabarcoding of the rRNA gene sequences [2]. In this case, BI clade dominated in the Norwegian Sea (Salinity: 35‰) but its proportion gradually diminished, being replaced by the novel *Bathycoccus* clade along the Barents Sea, the. Kara Sea, and the Laptev Sea, where the general seawater salinity decreased (the lowest: 3.5‰).

Moreover, we fortuitously discovered a contig (Contig_k141_506314, 4204 bp, GenBank accession: PP409568, Table S8) in one Baltic Sea metagenomic assembly (MA1, Table S5) that shared high similarity to the rRNA gene sequence from the Russian Arctic Seas (GenBank accession: MT648817.1), both of which were placed within the same novel clade in the phylogenetic tree of ITS1-5.8S-ITS2 sequences (Fig. S1). However, this contig could not be clustered with any of the MAGs during the binning process, including the generated MAG of *Bathycoccus* (BAL_E1), which belonged to the putative novel clade. This inability to cluster is like due to the aforementioned differences discussed [3]. Taxonomic annotation of the MAGs and rRNA gene sequences indicated the absence of any other types of *Bathycoccus* MAGs and rRNA gene sequences in this assembly and binning. Therefore, it is highly likely that this MAG and this contig of rRNA gene sequences exclusively belong to the same clade.

To further confirm this association, we calculated the Pearson correlation between ITS RPKM and genome RPKM for the BI (as a control) and the putative novel clade in the Baltic Sea metagenomic samples (Fig. S4d,e). As expected, the ITS RPKM and genome RPKM of the already known clade BI showed high Pearson correlation (r = 0.71, *P* value = 1.75e^-11^). For the putative novel clade, the correlation between the ITS RPKM and genome RPKM even more positive (r = 0.81, *P* value < 2.2e^-16^).

Based on the above evidence, we can confidently conclude that the discovered novel MAGs and rRNA gene sequences belong to the same clade, which we name as BIV clade. In total, there are four identified *Bathycoccus* clades, namely, BI, BII, BIII and BIV.

**Method. S2 Nucleic acid extraction, sequencing, genome assembly and annotation**

**Nucleic acid extraction and whole genome sequencing**

We selected the *Bathycoccus* strain UST710 for whole-genome sequencing. Prior to DNA extraction, algal cultures were subjected to antibiotic treatment to reduce bacterial load. The cells were harvested by centrifugation at 5000 g for 10 min at 4 °C. For PacBio long-read sequencing, high-molecular-weight genomic DNA was extracted using a modified CTAB protocol [5]. The SMRTbell library was constructed using standard library preparation protocols and sequenced on the PacBio Sequel Platform (Novogene, China). For Illumina sequencing, DNA was extracted using the DNeasy Plant Pro Kit (Qiagen, Germany) according to the manufacturer’s instructions. The Illumina library was prepared using the NEBNext Ultra IIDNA Library Prep Kit (NEB, USA) and sequenced on a Novaseq 6000 System (Illumina) by Novogene, China .

RNA sequencing (RNA-seq) was used to enhance gene prediction accuracy. Independent cultures were grown synchronously under the standard growth conditions. To maximize the capture of expressed genes, cultures in the exponential growth phase were harvested at three-hour intervals throughout a diel cycle. Total RNA was extracted using the Direct-zol RNA MiniPrep kit (Zymo Research, USA). All RNA samples were pooled for sequencing. The RNA-seq library was constructed using the NEBNext Ultra RNA Library Prep Kit (NEB, USA) and sequenced a Novaseq 6000 System (Illumina) by Novogene, China.

***De novo* genome assembly**

The raw reads from Illumina sequencing were trimmed using Trimmomatic v.0.39 [6]. The PacBio subreads were corrected and trimmed using Canu v.2.2 [7]. *De novo* assembly was conducted with Illumina and PacBio reads using various assemblers, and the best assembly was generated by Flye v.2.9.1-b1780, with default parameters except “--iterations 3” [8], as it successfully assembled all chromosomes from telomere to telomere. To improve the consensus accuracy of the assembly, Racon v.1.5.0 [9] was applied for one-round error correction using PacBio long reads, followed by three iterations of polishing by Pilon v.1.24 [10] using Illumina short reads. The quality of the assemblies was evaluated throughout the process: (1) Genome assembly metrics were generated by QUAST v5.0.2 [11]. (2) Genome completeness was evaluated by BUSCO v.5.5.0 [12] with the Chlorophyta odb10 dataset. (3) All assemblies were manually checked in Geneious Prime (<https://www.geneious.com/>).

**Annotation of repetitive elements and endogenous viral elements identification**

Repetitive elements were annotated through a combination of *de novo* and homology-based strategies. Novel transposable elements (TEs) were annotated using the TransposonUltimate *reasonaTE* pipeline [13]. The results were merged and clustered into a non-redundant *de novo* TE library. Repetitive elements were then identified using homology-based search against both the *de novo* TE library and the Repbase database (http://www.girinst.org/repbase) using RepeatMasker v.4.1.2 [14]. Endogenous viral elements were identified using ViralRecall [15] with the GVOG database, and geNomad [16] with the geNomad database (version 1.2).

**Protein-coding gene prediction and functional annotation**

Protein-coding genes for the repeat-masked genome were predicted by integrating *ab initio*, homology, and transcript-based gene prediction approaches. For transcript-based prediction, the trimmed RNA-seq reads were mapped to the genome assembly using HISAT2 v.2.2.1 [17] with default parameters, and the alignments were assembled using Trinity v.2.13.2 [18] and StringTie v.2.2.1 [19] under both *de novo* and genome-guided modes. The PASA pipeline v.2.5.2 [20] was used to merge all transcriptome assemblies, and TransDecoder v.5.7.0 (https://github.com/TransDecoder) was then used to predict open reading frames (ORF) on the PASA assembly. An *ab initio* gene prediction was performed using the BRAKER2 pipeline v.2.1.6 [21] for the soft-masked genome, incorporating extrinsic evidence from RNA-Seq and protein information. Homology-based prediction was conducted by querying the genome assembly against a custom Mamiellophyceae protein database using miniport [22]. All gene models were consolidated into a non-redundant gene set with EvidenceModeler EVM, v.2.0.0 [23] using a weight set as follows: homology, 5; transcript-based, 10; *ab initio*, 3; other (TransDecoder), 6. Functional annotation of the predicted gene set was performed using BLASTP or HMMER with an e-value of 10^−5^ against several known databases, including Swiss-Prot, NCBI NR, Pfam, EggNOG, GO and KEGG.

**References**

1. Majaneva M, Rintala J-M, Piisilä M, Fewer DP, Blomster J. Comparison of wintertime eukaryotic community from sea ice and open water in the Baltic Sea, based on sequencing of the 18S rRNA gene. *Polar Biol* 2012; **35**: 875–889.

2. Belevich TA, Milyutina IA, Abyzova GA, Troitsky AV. The pico-sized Mamiellophyceae and a novel Bathycoccus clade from the summer plankton of Russian Arctic Seas and adjacent waters. *FEMS Microbiology Ecology* 2021; **97**: fiaa251.

3. Mise K, Iwasaki W. Unexpected absence of ribosomal protein genes from metagenome-assembled genomes. *ISME COMMUN* 2022; **2**: 1–9.

4. Alneberg J, Sundh J, Bennke C, Beier S, Lundin D, Hugerth LW, et al. BARM and BalticMicrobeDB, a reference metagenome and interface to meta-omic data for the Baltic Sea. *Sci Data* 2018; **5**: 180146.

5. Craig RJ, Hasan AR, Ness RW, Keightley PD. Comparative genomics of Chlamydomonas. *The Plant Cell* 2021; **33**: 1016–1041.

6. Bolger AM, Lohse M, Usadel B. Trimmomatic: a flexible trimmer for Illumina sequence data. *Bioinformatics* 2014; **30**: 2114–2120.

7. Koren S, Walenz BP, Berlin K, Miller JR, Bergman NH, Phillippy AM. Canu: scalable and accurate long-read assembly via adaptive k-mer weighting and repeat separation. *Genome Res* 2017; **27**: 722–736.

8. Kolmogorov M, Yuan J, Lin Y, Pevzner PA. Assembly of long, error-prone reads using repeat graphs. *Nat Biotechnol* 2019; **37**: 540–546.

9. Vaser R, Sović I, Nagarajan N, Šikić M. Fast and accurate de novo genome assembly from long uncorrected reads. *Genome Res* 2017; **27**: 737–746.

10. Walker BJ, Abeel T, Shea T, Priest M, Abouelliel A, Sakthikumar S, et al. Pilon: An Integrated Tool for Comprehensive Microbial Variant Detection and Genome Assembly Improvement. *PLOS ONE* 2014; **9**: e112963.

11. Gurevich A, Saveliev V, Vyahhi N, Tesler G. QUAST: quality assessment tool for genome assemblies. *Bioinformatics* 2013; **29**: 1072–1075.

12. Simão FA, Waterhouse RM, Ioannidis P, Kriventseva EV, Zdobnov EM. BUSCO: assessing genome assembly and annotation completeness with single-copy orthologs. *Bioinformatics* 2015; **31**: 3210–3212.

13. Riehl K, Riccio C, Miska EA, Hemberg M. TransposonUltimate: software for transposon classification, annotation and detection. *Nucleic Acids Research* 2022; **50**: e64.

14. Tarailo-Graovac M, Chen N. Using RepeatMasker to identify repetitive elements in genomic sequences. *Curr Protoc Bioinformatics* 2009; **Chapter 4**: 4.10.1-4.10.14.

15. Aylward FO, Moniruzzaman M. ViralRecall—A Flexible Command-Line Tool for the Detection of Giant Virus Signatures in ‘Omic Data. *Viruses* 2021; **13**: 150.

16. Camargo AP, Roux S, Schulz F, Babinski M, Xu Y, Hu B, et al. You can move, but you can’t hide: identification of mobile genetic elements with geNomad. 2023. bioRxiv. , 2023.03.05.531206

17. Kim D, Paggi JM, Park C, Bennett C, Salzberg SL. Graph-based genome alignment and genotyping with HISAT2 and HISAT-genotype. *Nat Biotechnol* 2019; **37**: 907–915.

18. Haas BJ, Papanicolaou A, Yassour M, Grabherr M, Blood PD, Bowden J, et al. De novo transcript sequence reconstruction from RNA-seq using the Trinity platform for reference generation and analysis. *Nat Protoc* 2013; **8**: 1494–1512.

19. Pertea M, Pertea GM, Antonescu CM, Chang T-C, Mendell JT, Salzberg SL. StringTie enables improved reconstruction of a transcriptome from RNA-seq reads. *Nat Biotechnol* 2015; **33**: 290–295.

20. Bj H, Al D, Sm M, Jr W, Rk S, Li H, et al. Improving the Arabidopsis genome annotation using maximal transcript alignment assemblies. *Nucleic acids research* 2003; **31**.

21. Brůna T, Hoff KJ, Lomsadze A, Stanke M, Borodovsky M. BRAKER2: automatic eukaryotic genome annotation with GeneMark-EP+ and AUGUSTUS supported by a protein database. *NAR Genomics and Bioinformatics* 2021; **3**.

22. Li H. Protein-to-genome alignment with miniprot. *Bioinformatics* 2023; btad014.

23. Bj H, Sl S, W Z, M P, Je A, J O, et al. Automated eukaryotic gene structure annotation using EVidenceModeler and the Program to Assemble Spliced Alignments. *Genome biology* 2008; **9**.
